# Relative persistence of AAV serotype 1 vector genomes in dystrophic muscle

**DOI:** 10.1186/1479-0556-6-14

**Published:** 2008-10-15

**Authors:** Christina A Pacak, Thomas Conlon, Cathryn S Mah, Barry J Byrne

**Affiliations:** 1 Powell Gene Therapy Center, University of Florida, Gainesville, FL, USA; 2 Department of Pediatrics, University of Florida, Gainesville, FL, USA; 3 Division of Cellular and Molecular Therapy, Department of Pediatrics, University of Florida, Gainesville, FL, USA

## Abstract

The purpose of this study was to assess the behavior of pseudotyped recombinant adeno-associated virus type 1 (rAAV2/1) vector genomes in dystrophic skeletal muscle. A comparison was made between a therapeutic vector and a reporter vector by injecting the hindlimb in a mouse model of Limb Girdle Muscular Dystrophy Type 2D (LGMD-2D) prior to disease onset. We hypothesized that the therapeutic vector would establish long-term persistence through prevention of myofiber turnover. In contrast, the reporter vector genome copy number would diminish over time due to disease-associated muscle degradation.

One day old alpha sarcoglycan knockout mice (*sgca*^-/-^) were injected with 1 × 10^11 ^vector genomes of rAAV2/1-tMCK-*sgca *in one hindlimb and the same dose of rAAV2/1-tMCK-*LacZ *in the contra lateral hindlimb. Newborn mice are tolerant of the foreign transgene allowing for long-term expression of both the marker and the therapeutic gene in the null background. At 2 time-points following vector administration, hindlimb muscles were harvested and analyzed for LacZ or sarcoglycan expression. Our data demonstrate prolonged vector genome persistence in skeletal muscle from the hindlimbs injected with the therapeutic transgene as compared to hindlimbs injected with the reporter gene. We observed loss of vector genomes in skeletal muscles that were there were not protected by the benefits of therapeutic gene transfer. In comparison, the therapeutic vector expressing sarcoglycan led to reduction or elimination of myofiber loss. Mitigating the membrane instability inherent in dystrophic muscle was able to prolong the life of individual myofibers.

## Findings

Limb Girdle Muscular Dystrophy Type 2D (LGMD-2D) is an autosomal recessive disorder caused by mutations in the alpha sarcoglycan gene (*sgca*) and is the most prevalent of the sarcoglycanopathies; a class of dystrophies in which one of 6 transmembrane sarcoglycan proteins is deficient [1]. LGMD-2D affects both genders equally with onset typically occurring in the first decade of life [2]. The degree of severity in disease phenotype correlates with the amount of sgca protein present in the affected individual [3]. Presently, there is no definitive treatment available for this disease and care is aimed at minimizing disease progression. A clinically applicable gene delivery technique for LGMD-2D is being pursued by various investigators as a method for halting the debilitating consequences of sarcoglycan deficiency and similar diseases [4,5].

Adeno-associated virus (AAV) is a useful vehicle for gene transfer to skeletal muscle where it has been shown to persist as an episome [6,7]. Here we sought to examine the persistence of AAV genomes in dystrophic muscle over time. To do so, we injected the skeletal muscles of 4 one-day old alpha-sarcoglycan knockout (*sgca*^-/-^) mouse hindlimbs with 1 × 10^11 ^vector genomes of a vector we have previously shown to be therapeutic: rAAV2/1-tMCK-*sgca *[4]. This vector contains the human alpha sarcoglycan (*sgca*) gene and a previously described truncated murine creatine kinase (tMCK) promoter [4]. The skeletal muscles of the contra-lateral hindlimb were injected with 1 × 10^11 ^vector genomes of a reporter-gene containing vector: rAAV2/1-tMCK-*LacZ *which contains the same promoter driving the beta galactosidase (*LacZ*) gene. Neonatal mice were anesthetized by induced hypothermia and a 29.5-G tuberculin syringe was used to perform single intra-muscular (IM) injections of each vector formulated in phosphate-buffered saline (total volume of 35 μL per injection). The bevel of the needle was inserted facing up near the tendons of the anterior compartment at the ankle and pointing up into the tibialis anterior along the tibia into the upper hindlimb area. The virus solution was injected while withdrawing the needle to maximize area over which the vector was distributed.

At either 4 or 12 months post-administration, muscles were harvested and vector genomes were quantified and compared. Genomic DNA was isolated from frozen tissue samples as previously described [8]. The persistence of vector genomes was determined using the following PCR primer/probe set against the murine creatine kinase (tMCK) promoter. Forward Primer: 5'-GGCACCTATTGGTCTTACTGACA TC-3' Reverse Primer: 5'-GAGTGTCTCAGCCATGGTGGTA-3' Probe: 6FAM-CT CTCCACAGGTGTCCACTCCCAGTTCA-TAMRA.

**Figure 1:**
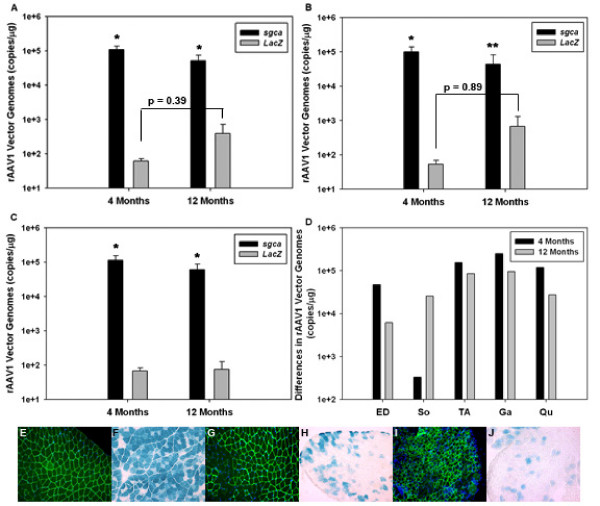
**Vector genome persistence**. (A-C) Log graphs showing vector genome amounts in individual muscles of the lower *sgca*^-/- ^mouse hindlimb at 4 or 12 months post administration of either rAAV2/1-tMCK-*sgca *(black bars) or rAAV2/1-tMCK-*LacZ *(grey bars). Greater persistence of vector genomes is observed in the *sgca *injected muscles (* indicates statistical significance [p-value ≤ 0.05], ** indicates p-value = 0.29). (A) Data for all muscles combined from the right (*sgca *injected) and the left (*LacZ *injected) hindlimbs at 4 or 12 months post injection. Muscles analyzed include: *extensor digitorum longus *(ED), *gastrocnemius *(Ga), *soleus *(So), *tibialis anterior *(TA), and *quadriceps *(Qu). (B) Combined (primarily) fast-twitch muscle data (ED and TA) at 4 and 12 months post injection. (C) Combined mixed/slow-twitch muscle data (Ga, So, and Qu) at 4 and 12 months post injection. (D) Bar graph depicting the differences in vector genome copy numbers in individual muscles at either 4 months (black) or 12 months (grey) post injection. Differences in expression levels between the two constructs were greater at 4 than at 12 months post administration. (E) Immunofluorescence image of a *quadriceps *muscle cryosection (2 months post rAAV2/1-tMCK-*sgca *administration) showing alpha-sarcoglycan located at the cell membrane (green) and nuclei maintained in the cell periphery (DAPI stain-blue). (F) β-galactosidase stained *quadriceps *muscle cryosection (2 months post rAAV2/1-tMCK-*LacZ *administration) showing staining in transduced myofibers (blue). (G-H) Images of *extensor digitorum longus *muscles (4 months post delivery of *sgca *or *LacZ *[respectively]). (I-J) Images of *soleus *muscles (4 months post delivery of *sgca *or *LacZ *[respectively]).

Our results show that at both 4 and 12 months post virus administration there were a statistically significantly higher number of vector genomes present in those hindlimb muscles injected with rAAV2/1-tMCK-*sgca *than those injected with rAAV2/1-tMCK-*LacZ *(Figure 1A). At 2 months post administration differences in the number of transduced myofibers between each hindlimb (as demonstrated by immunohistochemistry and LacZ staining of frozen skeletal muscle cryosections to identify alpha sarcoglycan [green] and β-galactosidase [blue]) were subtle (Figure 1E–F) but increased over time (Figure 1G–J). Vector genome assessment of *extensor digitorum longus *(EDL), and the *tibialis anterior *(TA) were combined as they are both composed of predominately fast-twitch myofibers (Figure 1B). At 4 months post virus administration a significant difference in the ability of the two vectors to persist over time in dystrophic muscle became evident. This difference was still present but was not as profound at 12 months post injection since the total number of vector genomes in rAAV2/1-tMCK-*sgca *injected EDL, and TA muscles decreased over time.

In contrast, vector genome assessment of muscles composed of either primarily slow-twitch or mixed amounts of each fiber type (*gastrocnemius *[Ga]*, soleus *[So], and *quadriceps *[Qu]) showed a significant difference between the two vector's relative persistence at both time points (Figure 1C). Specifically, the number of vector genomes detected in the Ga muscles decreased only slightly and those detected in the So muscles of the same hindlimbs did not decrease over time.

The difference or spread in the numbers of detectable genomes in each muscle as compared to those in the same muscle of the contra lateral hindlimb revealed an interesting pattern. The mean number of genomes detected for each *LacZ *muscle at both time points was subtracted from the mean number of genomes detected for each *sgca *muscle at both time points to allow for a comparison of differences (or range in mean genome number) between individual muscles in hindlimbs over time. Of those muscles assessed in this study the So is composed of the highest percentage of slow twitch myofibers and it was the only muscle that demonstrated a larger spread between numbers of vector genomes at the 12 month time point than at the 4 month. The EDL however, is composed of predominately fast twitch myofibers. When compared to the other muscles in this study it showed the greatest decrease in spread between persistent vector genomes in muscles from each leg. Our results suggest a greater overall amount of muscle turnover in the EDL than in the other individual muscles we analyzed regardless of which vector was administered. This may suggest that fast-twitch (type II) fibers (of which the EDL is primarily composed of [≥ 97%][9]) have a tendency to turnover more rapidly than muscles composed of either a mixture of fiber types or of primarily slow twitch fibers (type I).

To our knowledge, an explanation for the random development of dystrophic lesions in both mouse models of muscular dystrophy as well as the humans suffering from this disease has not yet been presented. Our data provides further evidence that individual muscles in this disease model may not all have the same rate of myofiber turn-over leading to tissue fibrosis/necrosis.

The IM delivery method used in this study does not sufficiently deliver vector to every muscle fiber in an equal manner. Therefore, there are likely areas of protected muscle that do contain the therapeutic transgene as well as unprotected muscle areas that deteriorate over time and could eventually overwhelm the treated myofibers and could be another explanation for higher-level turnover in the EDL. Additionally, the *sgca *protein is membrane bound and not secreted, so transduction of one cell will not be a sufficient way to provide therapy to the surrounding area. Because our delivery method is simple and allows for a single injection to transduce multiple muscles to a high degree, it is a useful proof of concept technique. Use of alternative IV delivery methods could provide a more even biodistribution and may be more clinically applicable [5,10].

Our data demonstrate the ability of a therapeutic vector to maintain the integrity of transduced muscle fibers, and thereby lead to improved myofiber survival. Our results derived from muscles transduced with a reporter gene vector serve as a model for determination of the amount of muscle fiber turnover between various muscles in this disease model. Future studies demonstrating protection of muscle from a degenerative disease could incorporate a cell proliferation assay such as that developed by Salic et al to further uncover the [11]. While the body's natural muscle regeneration machinery attempts to restore damaged tissue, the non-therapeutic (reporter gene) AAV genomes that would persist in normal muscle as episomes are lost in proportion to the diseased myofiber number. The ability of the rAAV2/1-tMCK-*sgca *vector to prevent myofibers from membrane damage has the potential to serve as an important therapeutic strategy in the future. Further studies of the potential for this vector to promote repair in muscle with existing dystrophy will be needed.

## Abbreviations

AAV: adeno-associated virus; ED: *extensor digitorum longus*; Ga: *gastronemius*; IM: intra-muscular; LacZ: beta galactosidase; LGMD-2D: limb girdle muscular dystrophy type 2D; PCR: polymerase chain reaction; Qu: *quadriceps*; sgca: alpha-sarcoglycan; So: *soleus*; TA: *tibialis anterior*; tMCK: truncated murine creatine kinase.

## Competing interests

The Johns Hopkins University, the University of Florida, and B.J.B. could be entitled to patent royalties for inventions related to the findings in this article.

## Authors' contributions

CAP participated in the design of the study, performed the injections, harvested the tissues, performed the immunohistochemistry analysis and drafted the manuscript. CSM participated in the design of the study and helped to draft the manuscript. TJC participated in the design of the study and performed vector genome analysis and helped to draft the manuscript. BJB participated in the design of the study. All authors read and approved the final manuscript.

## References

[B1] Moore SA, Shilling CJ, Westra S, Wall C, Wicklund MP, Stolle C, Brown CA, Michele DE, Piccolo F, Winder TL (2006). Limb-girdle muscular dystrophy in the United States. J Neuropathol Exp Neurol.

[B2] Kang PBKL, Scriver CRBA, Valle D, Sly WS (2007). Chapter 216: The Muscular Dystrophies. The Online Metabolic and Molecular Bases of Inherited Disease.

[B3] Eymard B, Romero NB, Leturcq F, Piccolo F, Carrie A, Jeanpierre M, Collin H, Deburgrave N, Azibi K, Chaouch M (1997). Primary adhalinopathy (alpha-sarcoglycanopathy): clinical, pathologic, and genetic correlation in 20 patients with autosomal recessive muscular dystrophy. Neurology.

[B4] Pacak CA, Walter GA, Gaidosh G, Bryant N, Lewis MA, Germain S, Mah CS, Campbell KP, Byrne BJ (2007). Long-term Skeletal Muscle Protection After Gene Transfer in a Mouse Model of LGMD-2D. Mol Ther.

[B5] Fougerousse F, Bartoli M, Poupiot J, Arandel L, Durand M, Guerchet N, Gicquel E, Danos O, Richard I (2007). Phenotypic Correction of alpha-Sarcoglycan Deficiency by Intra-arterial Injection of a Muscle-specific Serotype 1 rAAV Vector. Mol Ther.

[B6] Kessler PD, Podsakoff GM, Chen X, McQuiston SA, Colosi PC, Matelis LA, Kurtzman GJ, Byrne BJ (1996). Gene delivery to skeletal muscle results in sustained expression and systemic delivery of a therapeutic protein. Proc Natl Acad Sci USA.

[B7] Schnepp BC, Clark KR, Klemanski DL, Pacak CA, Johnson PR (2003). Genetic fate of recombinant adeno-associated virus vector genomes in muscle. J Virol.

[B8] Pacak CA, Mah CS, Thattaliyath BD, Conlon TJ, Lewis MA, Cloutier DE, Zolotukhin I, Tarantal AF, Byrne BJ (2006). Recombinant adeno-associated virus serotype 9 leads to preferential cardiac transduction in vivo. Circ Res.

[B9] Warren GL, Ingalls CP, Armstrong RB (2002). Temperature dependency of force loss and Ca(2+) homeostasis in mouse EDL muscle after eccentric contractions. Am J Physiol Regul Integr Comp Physiol.

[B10] Arruda VR, Stedman HH, Nichols TC, Haskins ME, Nicholson M, Herzog RW, Couto LB, High KA (2005). Regional intravascular delivery of AAV-2-F.IX to skeletal muscle achieves long-term correction of hemophilia B in a large animal model. Blood.

[B11] Salic A, Mitchison TJ (2008). A chemical method for fast and sensitive detection of DNA synthesis in vivo. Proc Natl Acad Sci USA.

